# Phloem-Localized Insect-Transmitted Bacteria Associated with Plant Diseases

**DOI:** 10.3390/microorganisms11102494

**Published:** 2023-10-05

**Authors:** Assunta Bertaccini

**Affiliations:** Department of Agricultural and Food Sciences, *Alma Mater Studiorum*, University of Bologna, 40127 Bologna, Italy; assunta.bertaccini@unibo.it

In the last three decades, an increasing number of plant diseases associated with the presence of phloem-localized insect-transmitted bacteria have been observed around the world, causing serious economic losses. Plant pathogens such as ‘*Candidatus* Phytoplasma’ species [1,2], ‘*Candidatus* Liberibacter asiaticus’, ‘*Candidatus* Liberibacter americanum’, ‘*Candidatus* Liberibacter africanus’ [3,4], and ‘*Candidatus* Liberibacter solanacearum’ [5] are responsible for serious damages to agricultural crops, landscape trees and shrubs, and forestry worldwide. It is, therefore, of the utmost relevance to fill gaps in the knowledge of their epidemiology and search for eco-friendly management strategies to reduce the impact of the diseases associated with these bacteria, to help the agricultural/forestry sector to become more sustainable, productive, and healthy, and also to reduce general environmental problems linked to the presence of these microorganisms in plants. Due to the recent limitations on research caused by the COVID-19 pandemic, many young scientists are unaware of the dangers posed by the infectious diseases, which continue to threaten and devastate large regions across the globe. This issue is also severe as these diseases and their associated pathogens and insect vectors exhibit a high propensity to spread into new areas due to climate change, their intrinsic ecology/biology, changes in agriculture or forestry management practices, and globalization. The most well-known plant diseases are, among others, citrus “huanglongbing”, potato zebra chips, “stolbur”, coconut lethal yellowing and grapevine “flavescence dorée”, all of which have serious economic impacts. For none of these disease have the Koch postulates been fulfilled and, therefore, the associated bacteria cannot be claimed to be etiological agents of the respective diseases. It is therefore critical to achieve this information confirmed to carry out an appropriate and well focused management. However, growing statistical evidence, supported by both consistent symptomatology and the consistent detection of the same microorganisms in the symptomatic plants of diverse species, is clearly indicating their relevant role in the diseases to which they are associated. Several reviews have described achievements in this field, but the basic biological knowledge for this microorganism has still not been sufficiently described in the literature. One of the main constraints in this field is the lack of knowledge about biological bases of interaction among plants, insect vectors and bacteria, which has made it very difficult to distinguish and appropriately manage the diverse diseases. Young scientists especially are not aware of the presence and the risks of these diseases that are infecting and very often destroying huge areas worldwide. This is especially important since they are seriously impacting very genetically homogeneous plant crops in both tropical and temperate areas due to the increasing impact of climate change. It would be, therefore, very useful if scientists from previous generations who have deeply studied these diseases could summarize their knowledge and transfer it to the new generations of scientists, in order to avoid the repetition of mistakes due to the loss of knowledge on a previous background. The web-based literature, whilst also a powerful tool, has caused the erasure of many historical records before the year 2000, despite this context being very useful to understand situations that are still very relevant to epidemic outbreaks. History is always the best teacher, especially in the never-ending battle to produce healthy and fruitful plants with a reduced impact of pesticides. Bacteria transmitted by flying insects and infecting immobile plants are serious and difficult constraints to worldwide agriculture.

All the challenges and potential recent and past knowledge related to these diseases and their associated agents are welcomed in the current Special Issue, entitled “Phloem-Localized Insect- Transmitted Bacteria Associated with Plant Diseases”, to analyze information, develop solutions, support the future research of young plant pathologists, and provide basic information for management of agriculturally safe and healthy crops worldwide.
